# Liver Transplantation Utilizing Mixed Biologic and Synthetic Arterial Conduits

**DOI:** 10.1155/2016/9245079

**Published:** 2016-10-12

**Authors:** Marcio F. Chedid, Tomaz J. M. Grezzana-Filho, Aljamir D. Chedid, Luiz Pedro P. Hendges, Ian Leipnitz, Mario R. Alvares-da-Silva, Ariane N. Backes, Matheus J. Reis, Cleber Dario P. Kruel, Cleber R. P. Kruel

**Affiliations:** ^1^Liver and Pancreas Transplant and Hepatobiliary Surgery Unit, Hospital de Clinicas de Porto Alegre, Federal University of Rio Grande do Sul (UFRGS), Porto Alegre, RS, Brazil; ^2^Division of Gastroenterology and Hepatology, Hospital de Clinicas de Porto Alegre, Federal University of Rio Grande do Sul (UFRGS), Porto Alegre, RS, Brazil

## Abstract

Arterial conduits are necessary in nearly 5% of all liver transplants and are usually constructed utilizing segments of donor iliac artery. However, available segments of donor iliac artery may not be lengthy enough or may not possess enough quality to enable its inclusion in the conduit. Although there are few reports of arterial conduits constructed solely utilizing prosthetic material, no previous reports of conduits composed of a segment of donor iliac artery and prosthetic material (mixed biologic and synthetic arterial conduits) were found in the medial literature to date. Two cases reporting successful outcomes after creation of mixed biologic and prosthetic arterial conduits are outlined in this report. Reason for creation of conduits was complete intimal dissection of the recipient's hepatic artery in both cases. In both cases, available segments of donor iliac artery were not lengthy enough to bridge infrarenal aorta to porta hepatis. Both patients have patent conduits and normally functioning liver allografts, respectively, at 4 and 31 months after transplant. Mixed biologic and synthetic arterial conduits constitute a viable technical option and may offer potential advantages over fully prosthetic arterial conduits.

## 1. Introduction

Arterial conduits are necessary in nearly 5% of all liver transplants [1, 2]. A segment of donor iliac artery usually is utilized as the arterial conduit, bridging recipient's infrarenal aorta to hepatic artery of the liver allograft (HAA) [3, 4]. However, available segments of donor iliac artery may not be lengthy enough or may not possess enough quality to enable its inclusion in the arterial conduit.

Although there are few reports of arterial conduits constructed solely utilizing prosthetic material [4, 5], no previous report of conduits composed of a segment of donor iliac artery and prosthetic material (mixed biologic and synthetic arterial conduits, MAC) has been found in the literature. The first report of successful utilization of MAC in liver transplantation is outlined in this report.

## 2. Methods

Reason for creation of conduits was complete intimal dissection of the recipient's hepatic artery in both cases reported herein. Arterial conduits solely utilizing segments of donor iliac artery to bridge infrarenal aorta to HAA were planned. However, available segments of donor iliac artery were not lengthy enough to reach porta hepatis.

## 3. Results

### 3.1. Case 1

**Figure 1:**
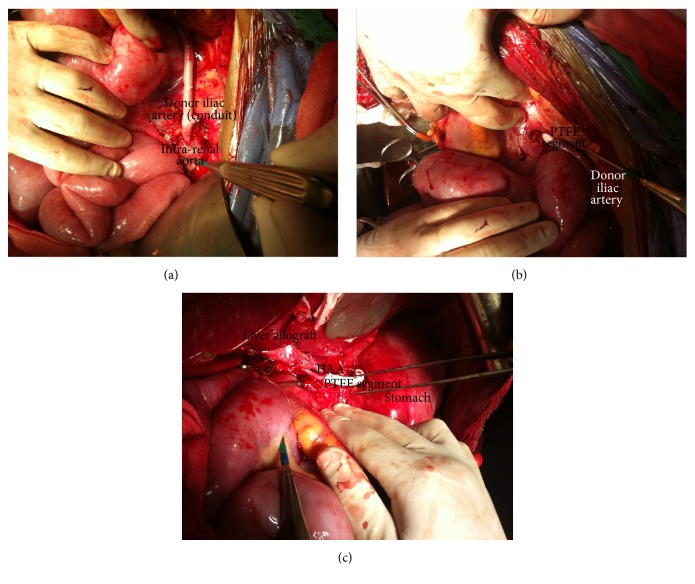
Creation of a MAC. (a) Anastomosis of proximal end of donor iliac artery to infrarenal aorta. (b) Anastomosis of distal end of donor iliac artery segments to a PTFE graft with retrocolic and retrogastric placement of the conduit. (c) Anastomosis of the distal end of PTFE graft to HAA.

A 51-year-old female underwent deceased donor liver transplant for HCV-related cirrhosis and hepatocellular carcinoma within Milan criteria. Available segment of donor iliac artery was sewn to recipient's infrarenal aorta utilizing running 5-0 polypropylene sutures (Figure 1(a)).

The conduit was placed prepancreatic and retrogastric, and the segment of donor iliac artery was sewn to a 6 mm polytetrafluoroethylene (PTFE) graft (Figure 1(b)).

Distal end of PFTE graft was sewn to Hepatic Artery of the Allograft (HAA) utilizing running 7 mm polypropylene sutures (Figure 1(c)).

Patient was maintained on intravenous heparin for 72 hours and transitioned to oral acid acetylsalicylic 100 mg/day thereafter. Complications included urinary sepsis and transient neurologic toxicity to tacrolimus. She was discharged home on posttransplant day 25. MAC is patent and allograft has normal function 31 months after transplant.

### 3.2. Case 2

A 61-year-old female also underwent deceased donor liver transplant for HCV-related cirrhosis and hepatocellular carcinoma within Milan criteria. A MAC was created through sewing a number 8 PTFE graft to recipient's infrarenal aorta utilizing 4-0 running polypropylene sutures. Distal end of PTFE graft was sewn to the available segment of donor iliac artery utilizing running 5-0 polypropylene sutures. Distal end of donor iliac artery was sewn to HAA utilizing running 7-0 sutures. Patient was maintained on intravenous heparin. Posttransplant complications included transient acute renal failure and reoperations for intraperitoneal hemorrhage on posttransplant day 8 and for fascial dehiscence on day 27. Following reoperation for intraperitoneal bleeding, heparin was stopped, and patient was switched to oral acid acetylsalicylic 100 mg/day. She was discharged home with patent arterial conduit and normal allograft function on posttransplant day 57. She currently has a patent MAC and normal liver allograft function at 4 months after transplant.

## 4. Discussion

Nikitin et al. reported similar long-term survival for LT utilizing arterial conduits as compared to LT employing standard arterial reconstructions [1]. Although LT using arterial conduits can be performed safely in experienced hands, arterial conduits carry a potential for increased posttransplant complications and also higher rates of graft loss. Hibi et al. [2] and Liu et al. [5] reported increased rates of complications and decreased graft survival for LT utilizing as compared to LT utilizing standard arterial reconstructions [2, 5]. Potential complications related to arterial conduits include an increased risk of hepatic artery thrombosis.

Arterial conduits were utilized in 6 out of our last 150 LTs. In the two cases reported herein, segments of donor iliac artery were not lengthy enough to reach HAA. Instead of utilizing a full synthetic graft, MACs utilizing the available segment of donor iliac artery and a shorter segment of a PTFE graft were created. Although utilization of MACs could have been performed elsewhere before, the authors did not find any previous report on such technique in the medical literature. Thus, to our knowledge, this is the first report of LT utilizing MAC. In both cases, prevention of arterial thrombosis was performed through initial intravenous heparin drip, both patients being transitioned to oral salicylic acid during the first ten posttransplant days.

As utilization of prosthetic material may confer additional risks to those of conduits solely utilizing vessels from a deceased donor, there is reluctance across transplant surgeons to use prosthetic conduits in abdominal transplant recipients. Potential risks of prosthetic arterial conduits include increased potential for abdominal sepsis secondary to infection of the prosthetic graft. Thus, the literature reports of arterial conduits utilizing prosthetic material are scarce [6]. Such reports on LT utilizing prosthetic conduits usually include single or very few cases reports included in larger series reporting on outcomes of conduits utilizing solely donor vessels [4, 5]. As happened to the cases reported herein, the authors believe that prosthetic material only should be employed for construction of arterial conduits in the rare instances in which appropriate donor vessels are unavailable.

Although conduits may be related to worse outcomes after LT [2, 5], outcomes of the cases reported herein were favorable. Further reports are necessary to evaluate utilization of MACs large scale and also to determine whether MACs would offer any advantage over fully prosthetic arterial conduits.

## Abbreviations

HAA: Hepatic artery of the liver allograft
MAC: Mixed biologic and synthetic arterial conduits
PTFE: Polytetrafluoroethylene.
